# Uncovering Active Constituents Responsible for Different Activities of Raw and Steamed *Panax notoginseng* Roots

**DOI:** 10.3389/fphar.2017.00745

**Published:** 2017-10-18

**Authors:** Yin Xiong, Lijuan Chen, Yupiao Hu, Xiuming Cui

**Affiliations:** ^1^Faculty of Life Science and Technology, Kunming University of Science and Technology, Kunming, China; ^2^Yunnan Key Laboratory of Panax notoginseng, Kunming, China; ^3^Laboratory of Sustainable Utilization of Panax notoginseng Resources, State Administration of Traditional Chinese Medicine, Kunming, China

**Keywords:** *Panax notoginseng*, bioactive constituents, anticoagulation, antioxidation, quality control

## Abstract

Although *Panax notoginseng* (PN) roots in raw and steamed forms were historically supposed to be different in the efficacies, the raw materials and steamed ones were often undifferentiated in the use and market circulation, which might bring unstable curative effects or even adverse reactions. To uncover chemical constituents responsible to different activities of raw and steamed PN, chemometrics analyses including partial least squares regression (PLSR) and multi-linear regression analysis (MLRA) were used to establish the relationships between the chromatographic fingerprints and activities of PN samples. Chemical fingerprints of PN were determined by HPLC. Anticoagulant and antioxidant activities were evaluated by the thromboplastin inhibiting test and hydroxyl radical scavenging assay, respectively. Results showed that there was a significant difference in the chemical composition between raw and steamed PN, which could be discriminated by principle component analysis according to different steaming temperatures. Compared with the steamed PN, raw PN exhibited stronger anticoagulation and weaker antioxidation. By chemometrics analyses, notoginsenoside R_1_, ginsenosides Rg_1_, Re, Rb_1_, and Rd were found to be the major active constituents of raw PN, whereas ginsenosides Rh_1_, Rk_3_, Rh_4_, and 20(*R*)-Rg_3_ had the key role in the activities of steamed PN, which could be used as new markers for the quality control (QC) of steamed PN.

## Introduction

The processing of herbal medicines, including special crafts of steaming, baking, cooking, and other methods with liquid or solid supplementary materials, plays an important role in the application of traditional Chinese medicine (TCM). The purposes of processing include transforming the properties of medicines, strengthening the curative efficacy, generating new effects and reducing the toxicity or side-effects (Wang et al., 2012; Li et al., 2013). In recent decades, it has been found that the main mechanism underlying the property changes of herbs is mainly related to the alteration in the chemical composition and/or bioactivity of constituents in herbs (Cao et al., 2011; Li S. L. et al., 2012). For example, diester diterpene alkaloids responsible for the toxicity of Aconitum Radix could be decomposed into less or non-toxic derivatives by boiling the raw materials at 100°C for 8 h before drying it (Sun et al., 2012). Thus, the uses of raw and processed medicines cannot be mixed up for different curative or toxic effects.

*Panax notoginseng* (PN) Burk., a plant in genus *Panax* (Araliaceae), is a well-known medicinal herb used to treat blood disorders for more than 400 years (Ge et al., 2015; Li et al., 2016). Based on the US Dietary Supplement Health and Education Act of 1944, PN and the relative products were also classified as dietary supplements (103rd Congress, 1994). Traditionally, there are two forms of PN, namely raw PN and steamed PN, of which the former one is used as a hemostatic, traumatic, and cardiovascular medicine removing blood stasis, while the steamed one is used as a tonic for nourishing the body and improving the health (Ge et al., 2015; Gu et al., 2015). Pharmacologic studies have also shown that the effects of PN changed when steamed. Lau et al. (2009) reported that the treatment of raw PN extract resulted in shorter bleeding time compared with rats treated with steamed PN, which is consistent with the traditional use of raw PN as a hemostatic. While for steamed PN, it could significantly increase the levels of hemoglobin and white blood cells, as well as the organ index of mice with blood deficiency caused by cyclophosphamide, which were inapparent when treated with raw PN (Zhou et al., 2014). Triterpenoid saponins including ginsenosides and notoginsenosides are considered to be the major bioactive constituents of PN, among which ginsenosides Rg_1_, Rb_1_, Re, and notoginsenoside R_1_ show higher levels than others in raw PN (Kim, 2012).

Despite the difference in traditional uses and pharmacological effects, trade in raw and steamed PN has still been complicated by the mixed use. Until now, only raw PN material or powder is recorded in the pharmacopeias of different countries or regions, in which the standard of processed PN is not included yet (British Pharmacopoeia Commission, 2014; Chinese Pharmacopoeia Commission, 2015; European Pharmacopoeia 8.0^1^; U.S.A Herbal Medicines Compendium 1.0^2^), indicating that the difference between the raw and steamed PN has not been authorized by codex standards. Even for some institution standards involving steamed PN, the marker constituents for its quality control (QC) are consistent with those of raw PN, despite the significant change in the chemical composition of PN during the steaming process reported in several studies (Wang et al., 2012; Ge et al., 2015). For example, in the China Food and Drug Administration (2012), the marker constituents for the QC of steamed PN powder are ginsenoside Rb_1_, ginsenoside Rg_1_, and notoginsenoside R_1_, which are same as the markers of raw PN involved in Chinese Pharmacopoeia of 2015 edition. Due to the lack of respective quality standards to differentiate raw and steamed PN, there would be risks of unstable curative effects or even adverse reactions for customers. Therefore, besides the investigation on changes in the chemical composition and pharmacological effects of PN during the steaming process, constituents responsible to different activities should also be uncovered for the individualized QC of raw and steamed PN, which have not been reported yet.

Since the traditional efficacy of removing blood stasis and promoting blood circulation of raw PN could be related to the anticoagulant effect (Li L. et al., 2012), and the body tonifying function of herbal medicines is partly attributed to their antioxidant and immunomodulatory effects by modern pharmacological researches (Yim and Ko, 2002), the anticoagulation (obtained by thromboplastin inhibiting test) and antioxidation (obtained by hydroxyl radical scavenging assay) activities of PN during the steaming process were studied in this research. Meanwhile, we developed the HPLC chromatographic fingerprints of PN under different steaming conditions, and investigated the correlation between the activities and fingerprints of PN samples by using multivariate regression techniques including principal component analysis (PCA), partial least squares regression (PLSR), and multi-linear regression analysis (MLRA). Constituents/peaks predicted to be responsible for different activities of PN were then identified, of which the activities were finally verified by pharmacologic tests.

## Materials and methods

### Chemicals

The reference standards of ginsenosides Rg_1_, Re, Rb_1_, Rd, Rh_1_, 20 (*R*)-Rg_3_, Rh_4_, Rk_3_, and notoginsenoside R_1_ were purchased from the National Institutes for the Control of Pharmaceutical and Biological Products (Beijing, China). Methyl alcohol and acetonitrile (HPLC grade) were purchased from Sigma-Aldrich, Inc. (St. Louis, MO, USA). Ultrapure water was generated with an UPT-I-20T ultrapure water system (Chengdu Ultrapure Technology, Inc., Chengdu, Sichuan, China). 1,1-diphenyl-2-picrylhydrazyl was purchased from the Sigma Chemical Co. (St. Louis, MO, USA). 1,10-phenanthroline, and L-ascorbic acid were purchased from the Xilong Chemical Co. (Guangzhou, Guangdong, China). All other chemicals used were of analytical grade.

### Sample preparation

Samples were obtained from a single batch of PN root in Yunnan, China. Steamed PN samples were prepared by steaming the crushed raw PN in an autoclave (Shanghai, China) for 2, 4, 6, 8, and 10 h at 105, 110, and 120°C, respectively. The steamed powder was then dried in a heating-air drying oven at about 45°C to constant weight, then powdered and sieved through a 40-mesh sieve.

### Animals

Kunming mice, male and female, weighing 18–22 g, were purchased from Tianqin Biotechnology Co. Ltd., Changsha, Hunan (SCXK (Xiang) 2014-0011). Before the experiments, the mice were given 1-week acclimation period in a laboratory at room temperature (20–25°C) and constant humidity (40–70%), and fed with standard rodent chow and tap water freely. Animal experimental procedures in the study were strictly conformed to the Guide for the Care and Use of Laboratory Animals and related ethics regulations of Kunming University of Science and Technology. The protocol was approved by the Experimental Animal Welfare and Ethics Committee, Kunming University of Science and Technology.

### HPLC analyses

The sample solutions were prepared according to the method described in Chinese Pharmacopoeia Commission (2015). HPLC analyses were done on an Agilent 1260 series system (Agilent Technologies, Santa Clara, CA, USA) consisting of a G1311B pump, a G4212B DAD detector and a G1329B autosampler. A Vision HT C_18_ column (250 × 4.6 mm, 5 μm) was adopted for the analyses. The mobile phase consisted of A (ultra pure water) and B (acetonitrile). The gradient mode was as follows: 0–20 min, 80% A; 20–45 min, 54% A; 45–55 min, 45% A; 55–60 min, 45% A; 60–65 min, 100% B; 65–70 min, 80% A; 70–90 min, 80% A. The flow rate was set at 1.0 mL/min. The detection wavelength was set at 203 nm. The column temperature was set at 30°C and sample volume was set at 10 μL.

### Anticoagulation test *in Vitro*

Blood was collected from healthy mice and directly transferred into citrated tubes (0.109 mol citrate, 9:1). The supernatant platelet-poor plasma (PPP) was obtained by centrifuging the blood samples above at 3,000 rpm for 10–15 min. The mixture of PPP and thrombokinase of various concentrations at the proportion of 2:1 (v/v) of total 50 μL was added into the test cup and incubated for 3 min at 37°C in a Blood Coagulation Instrument (XN06 series, Diagnostic Technology Ltd of Wuhan Jingchuan, China). 10 U/mL thrombokinase of 100 μL dissolved in 0.1 mol/L Tris-HCl buffer solution (pH 7.4) was subsequently added and incubated at the same condition. The prothrombin time (PT) was determined in accordance with the manufacturer's recommended protocols. The prolongation rate of *PT* was calculated according to the following equation:

(1) $$\text{Prolongation rate of }PT(\%)=(PT-PT_{0})/PT_{0}\times 100\%$$

where *PT*_0_ was the PT of control (blank, the normal saline replaced of thrombokinase), *PT* was the PT in the presence of thrombokinase.

The standard curve was drawn with the concentration of thrombokinase (*U*_*i*_) as the *X* axis and the lg [prolongation rate of *PT* (%)] as the *Y* axis. PN samples of 5 g, in the powdered form, were extracted with pure water (50.0 mL) by refluxing twice for 2 h at 80°C. The combined solution was filtered and concentrated under reduced pressure to the extract containing 0.1 g/mL of PN. The extract was then diluted with the normal saline to different concentrations. The prothrombin time of the mixed plasma sample containing PPP and PN extract (*PT'*) of different concentrations was determined. The prolongation rate of *PT'* was calculated according to the following equation:

(2) Prolongation rate of PT'(%)=(PT'−PT0')/PT0'×100%

where *PT*_0_' was the prothrombin time of control (blank, the normal saline replaced of extracts), *PT*' was the prothrombin time in the presence of extracts.

The corresponding concentration of thrombokinase (*U*_*i*_) was determined according to the standard curve. And the thromboplastin inhibition rate (%) was calculated according to the following equation:

(3) $$\begin{array}{c} \text{Thromboplastin inhibition rate}\;(\%)=(U_{i}-10)/10\times 100\% \end{array}$$

where *U*_*i*_ was the concentration of thromboplastin determined by the standard curve.

### Antioxidation test *in Vitro*

The extracts prepared in “Anticoagulation Test *in vitro*” were diluted with normal saline to 0.5, 1, 1.5, 2, 2.5, 3, and 3.5 mg/mL, respectively. The scavenging activity for hydroxyl radicals was measured according to the procedure described by Zhao et al. (2006). Reaction mixture contained 60 μL of 1.0 mmol FeCl_2_, 90 μL of 1 mmol 1,10-phenanthroline, 2.4 mL of 0.2 mol phosphate buffer (pH7.8), 150 μL of 0.17 mol H_2_O_2_, and 1.5 mL of extracts prepared. The reaction was started by adding H_2_O_2_. After incubation at room temperature for 5 min, the absorbance of the mixture at 560 nm was measured with a spectrophotometer. The scavenging activity for hydroxyl radicals was calculated according to the following equations:

(4) $$\begin{array}{c} \text{Scavenging rate}=[1-(A_{1}-A_{2})/A_{0}]\times 100\% \end{array}$$

Where *A*_0_ was the absorbance of the control, *A*_1_ was the absorbance in the presence of the extract and *A*_2_ was the absorbance without 1,10-phenanthroline.

### Pharmacologic verification

Constituents/peaks predicted to be responsible for different activities of PN were then identified by reference standards, of which the activities were finally verified by pharmacologic evaluation using the methods described in section “Anticoagulation Test *in vitro*” and “Antioxidation Test *in vitro*.”

### Statistical analyses

All data were expressed as means ± SD. SPSS 21.0 software (Statistical Program for Social Sciences, SPSS inc, Chicago) was applied to carry out the two-tailed unpaired *t*-test and PCA. DPS 9.50 software (Data Processing System, China) was used for MLRA. Umetrics SIMCA-P 11.5 software (Sartorius Stedim Biotech, Sweden) was applied for PLS analysis. A value of *P* < 0.05 was considered to be significant difference. A value of *P* < 0.01 was considered to be highly significant difference. EC_50_ value was fitted by Probit regression with Origin 7.5 for windows (OriginLab Corporation, USA) software.

## Theory

### PCA

PCA is applied for data compression and visualization. PCA produces so-called latent variables, here called principal components (PCs), which are linear combinations of the original manifest variables. The orthogonal PCs are constructed in such way that they maximize the description of the data variance in the *n* × *p* data matrix *X*. The projections of the objects (PN samples) on *PC*_*i*_ are the scores on *PC*_*i*_, and the projections of the variables (HPLC fingerprint signals here) on *PC*_*i*_ are the loadings on *PC*_*i*_. Thus, score plots give information related to the (dis)similarity of the objects, while on loading plots information about the contribution of the original variables to a given *PC*_*i*_ can be found (Nguyen Hoai et al., 2009).

### MLRA

MLRA attempts to model the relationship between two or more variables and a response by fitting a linear equation to observed data (Noori et al., 2010; Placca et al., 2010). The general purpose of MLRA is to learn about the relationship between several independent variables and a dependent variable. MLRA can be generally represented in the following form:

(5) $$\begin{array}{c} Y=b_{0}+b_{1}X_{1}+b_{2}X_{2}+b_{3}X_{3}+\ldots +b_{n}X_{n} \end{array}$$

where *Y* is the estimated value and represents the dependent variable. *X*_1_, *X*_2_, *X*_3_,…, *X*_n_ are measures of not correlated variables that may help in estimating *Y*. For example, *X*_1_ is the known score of the first independent variable, *X*_2_ is the known score of the second independent variable, etc. The coefficient *b*_0_ is the estimated constant, and *b*_1_, *b*_2_, *b*_3_…, *b*_n_ are called the regression coefficients (Hair et al., 1999).

### PLSR

PLSR is used to find the inner relationship between independent variables (*X*) and dependent variables (*Y*), which are simultaneously modeled by taking into account not only *X* variance, but the covariance between *X* and *Y* (Martens and Naes, 1989). In our study, the *X* matrix is composed of the enhanced fingerprints and the *Y* vector is constructed with the reference values of anticoagulation and antioxidation activities (EC_50_) obtained by the thromboplastin inhibition rate and hydroxyl radicals assay, respectively. Then, *X* and *Y* are decomposed in a product of another two matrices of scores and loadings; as described by the following equations:

(6) $$\begin{array}{c} X=TP^{T}+E \end{array}$$

(7) $$\begin{array}{c} Y=UQ^{T}+F \end{array}$$

where *TP*^*T*^ approximates to the chromatographic data and *UQ*^*T*^ to the true *Y* values; notice that the relationship between *T* and *U* scores is a summary of the relationship between X and Y. The terms *E* and *F* from the equations are error matrices. Hence, the PLS algorithm attempts to find factors (called Latent Variables) that maximize the amount of variation explained in *X* that is relevant for predicting *Y*; i.e., capture variance and achieve correlation (Brereton, 2007).

## Results

### HPLC fingerprints

**Figure 1:**
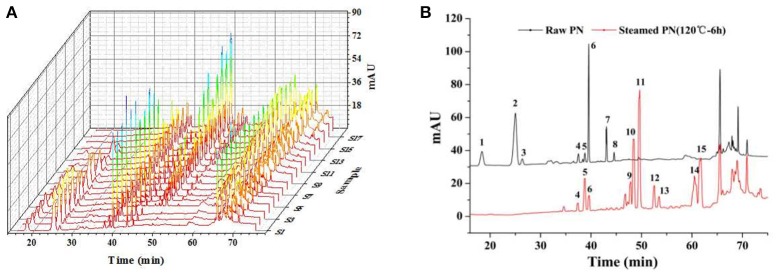
HPLC fingerprints **(A)** and the common peaks **(B)** of 18 batches of raw and steamed *Panax notoginseng* (PN) extracts. HPLC analyses were done on a Vision HT C_18_ column (250 × 4.6 mm, 5 μm) at 30°C. The mobile phase consisting of **(A)** (ultra pure water) and **(B)** (MeCN) was used at a flow rate of 1.0 ml/min as the following gradient mode: 0–20 min, 80% A; 20–45 min, 54% A; 45–55 min, 45% A; 55–60 min, 45% A; 60–65 min, 100% B; 65–70 min, 80% A; 70–90 min, 80% A. The detection wavelength was set at 203 nm and the injection column was set at 10 μL.

The results of methodology validation showed that the relative standard deviation values for precision, reproducibility and storage stability were less than 3.0, 4.0, and 3.0%, respectively. All the results indicated that the method of HPLC for the fingerprint analyses was valid and satisfactory. The optimized conditions for the 90-min HPLC fingerprints were described in sections “HPLC Analyses.” The chromatograms were generated for all batches of PN samples (Figure 1A), and for a typical raw PN sample and a steamed PN sample (Figure 1B). Peaks with good segregation, which also occupied large areas from consecutive peaks, were determined as the common peaks of PN samples. Therefore, fifteen peaks were selected by comparing their ultraviolet spectra and HPLC retention time. The method used to identify common peaks refers to reports in similar researches (Zheng et al., 2014; Shi et al., 2016). Along with the duration of steaming time and rise of temperature, the area and height of major peaks (peaks 1–3, and 6–8) in the raw PN were decreased gradually, while other peaks (peaks 5, 9–15) were increased or formed (Figure 1B).

The areas of 15 peaks in 18 batches of PN samples were listed in Table 1. The peak area was defined as 0 for peaks lacked in chromatograms. The coefficients of variance for almost all common peaks were higher than 46.6%. This is due to the diversity in the levels of constituents contained in samples under different process conditions. The areas of 15 common peaks were used for the following analysis.

**Table 1:** The samples information and peak area of fifteen common peaks in raw and steamed *Panax notoginseng* (PN) samples.

**Sample**	**NO.**	**Peak area of each peak**														
		**1^a^**	**2**	**3**	**4**	**5**	**6**	**7**	**8**	**9**	**10**	**11**	**12**	**13**	**14**	**15**
Raw PN 1	S1	360.8	1,348.8	98.2	93.2	85.7	727.1	229.3	59.7	0	0	0	0	0	0	0
Raw PN 2	S2	337.5	1,304.7	94.2	89.6	84.1	427.8	262.3	24.8	0	0	0	0	0	0	0
Raw PN 3	S3	560.6	1,435.1	108.4	173.2	173.8	1356.8	462.3	24.8	0	0	0	0	0	0	0
105°C-2 h	S4	430.4	1,347.6	68.5	109.1	161	828.6	246.5	42.1	29.2	66.1	133.1	0	0	0	56.6
105°C-4 h	S5	321.5	1,182.1	134.8	121.9	191.9	759.7	234.3	59.9	101.9	167.4	252.2	38.7	0	49.3	108.2
105°C-6 h	S6	251.4	1,003.3	94.8	113.6	221.1	831.5	290.5	97.4	106.9	266.1	423.1	76.6	38.9	58.5	215.1
105°C-8 h	S7	267	789.5	69.4	143.9	300.5	566.7	185	66.3	221.9	398.7	609	87.1	39.6	79.4	283.4
105°C-10 h	S8	199.8	685.2	78	147.8	341.9	704.8	226.3	92.2	230.8	465.1	709.7	125.1	53.4	110.9	373.6
110°C-2 h	S9	282.9	1,160	109.7	104.3	176.8	821.1	232.6	93.6	57.7	153.1	266	35.1	0	0	102
110°C-4 h	S10	201.4	1,021.8	74.8	85.5	22.9	687.6	207.5	99	97.5	308.5	521.9	87.8	31.6	0	229
110°C-6 h	S11	172.9	781	71.6	132	402.8	634.5	194.2	68.5	201.5	581.5	873.3	136.5	56.5	185.1	418.4
110°C-8 h	S12	89.7	369.4	0	145.1	422.8	440	136.6	37.3	293.5	761.2	1,136.9	173.9	79.8	256.9	559.7
110°C-10 h	S13	0	164.4	0	149.9	550.2	415.5	115.3	21.9	314.8	892.8	1,373.8	274.5	149.2	346.9	759.4
120°C-2 h	S14	127.3	629.9	79.4	106	349.1	505	161.8	27.5	169.8	571.1	800.2	134.9	62.9	200.8	421
120°C-4 h	S15	0	168.2	0	190.3	426.7	407	133.7	31.3	477.8	821.6	1,281.5	142.5	133.7	444.3	939.3
120°C-6 h	S16	0	0	0	110.3	474	249.1	52.8	0	331	1,030.7	1,641.7	279.6	135.6	709.8	1,012.9
120°C-8 h	S17	0	0	0	110.8	409.4	174.4	44	0	371.9	1,002.8	1,557.7	316.4	153.4	642.1	1,052.2
120°C-10 h	S18	0	0	0	97.1	230.6	196.8	0	0	368.3	1,058.7	1,748.3	293.5	150.7	532.3	1,056.4
C.V.^b^ (%)		81.1	67.4	75.5	23.1	52.4	46.6	53.9	70.2	76.7	77.6	78.0	85.6	95.6	114.7	88.8

a *Peaks 1–15*,

b *C.V. (%) = δ / μ × 100; δ is the standard deviation, μ is the average value of peak*.

### Clustering results by PCA

**Figure 2:**
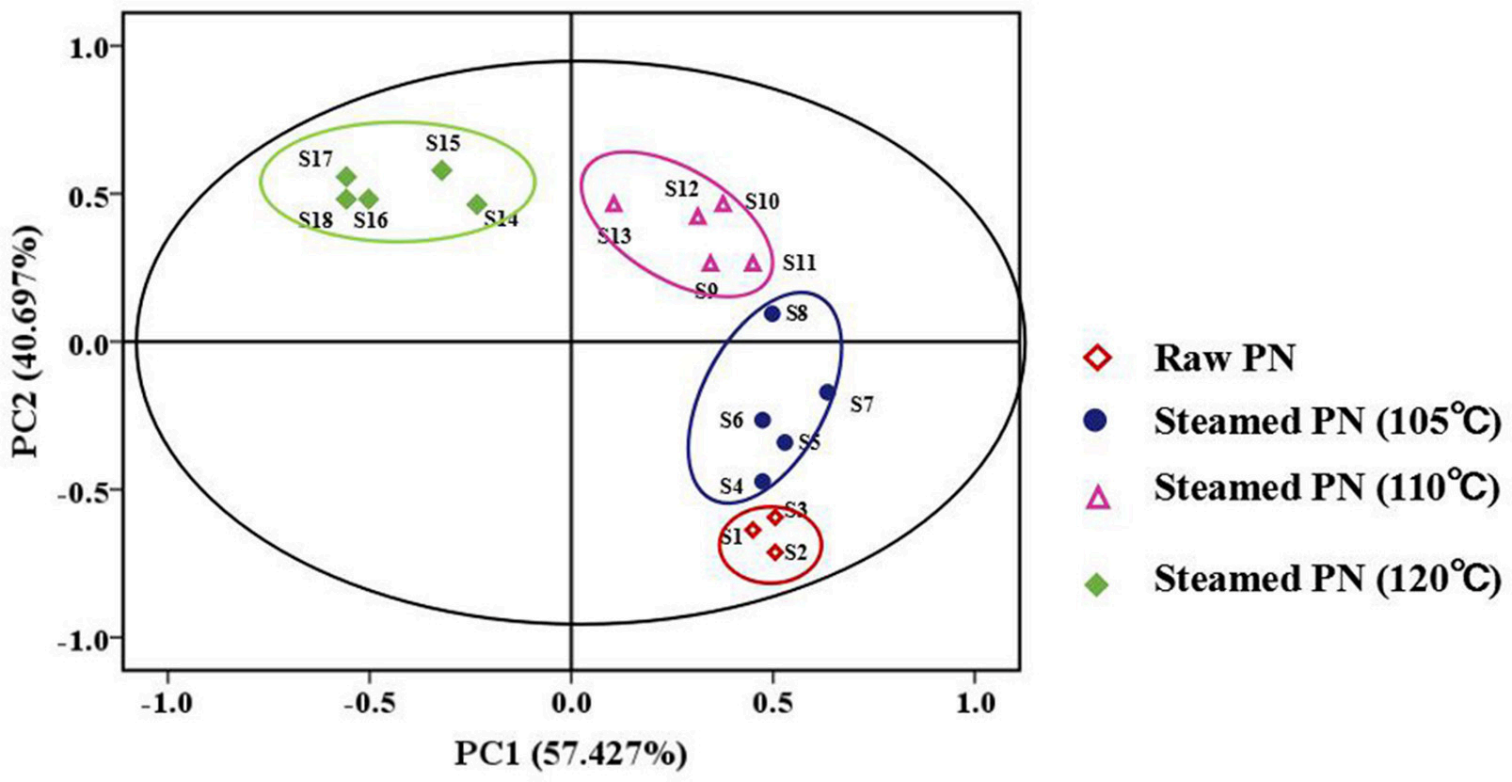
The clustering result of the fingerprints of *Panax notoginseng* (PN) samples by PCA.

PCA is a classical technique to reduce the dimensionality of the data set by transforming to a new set of variables, named PCs to summarize the features of the data. Since PCs are uncorrelated and ordered, the first few PCs, which contain most of the variations in the data, are usually used in cluster analysis (Yeung and Ruzzo, 2001). As shown in Figure 2, the fingerprints of PN samples were separated into four clusters according to the peak area. Samples 1–3 in cluster one were raw PN. Samples 4–8 in cluster two were PN steamed at 105°C. Samples 9–13 in cluster three were PN steamed at 110°C. Samples 14–18 in cluster four were PN steamed at 120°C. It indicated that PN samples steamed at the same temperature had similar chemical fingerprints. And PCA could initially separated raw and steamed PN samples at different temperatures from the chemical level. The results also suggested that compared with the steaming time, the steaming temperature had a more important role in the change of chemical composition of PN. Based on the results, a combination of PCA and HPLC methods could, at least roughly, discriminate raw and PN samples under different process conditions.

### Anticoagulation test

**Figure 3:**
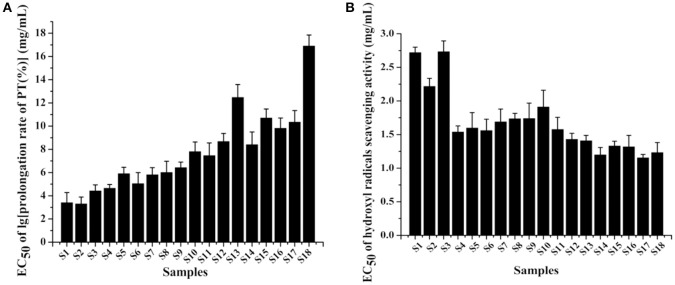
The anticoagulation activity **(A)** and antioxidation activity **(B)** of 18 batches of *Panax notoginseng* (PN) samples.

PT is used to evaluate the overall efficiency of extrinsic clotting pathway. A prolonged PT indicates a deficiency in coagulation factors V, VII, and X (Chan et al., 2007). In the study, the EC_50_ determined by the logarithm to base 10 of *PT* prolongation rate was applied to evaluate the anticoagulant effect of PN. The standard curve between the concentration of thrombokinase and logarithm of *PT* prolongation rate showed a good linearity (*R* = −0.9991). As shown in Figure 3A, raw PN samples (S1–S3) exhibited lower EC_50_ values of anticoagulation, suggesting that the anticoagulant effect of raw PN was stronger compared with steamed PN samples. The EC_50_ values of PN steamed at the same temperature were generally increased along with the increase of steaming time. For PN steamed for the same time, the higher the steaming temperature, the higher was the EC_50_ values of samples. Among these samples, S18 steamed for the longest time of 10 h at the highest temperature of 120°C showed the highest EC_50_ value, suggesting that its anticoagulant activity was the weakest compared with other ones.

### Antioxidation test

PN roots are mostly consumed as popular food tonic in the soup form by people in the southern region of China. Various studies have suggested that the tonifying functions of Chinese herbal medicines could be due to, at least partially, the protective effects against oxidation (Yim and Ko, 2002). Hydroxyl radical is very reactive which can be generated in biological cells through the Fenton reaction. Meanwhile, hydroxyl radical scavenging assay is commonly used for the determination of antioxidant activities of plant extracts. And PN showed higher sensitivity of scavenging hydroxyl radicals than other ones like 1,1-diphenyl-2-picrylhydrazyl free radicals in our previous work (Figure S1). Therefore, the method was applied to investigate the antioxidant effect of raw and steamed PN roots, with results shown in Figure 3B, where EC_50_ was the concentration of PN scavenging 50% hydroxyl radicals. According to the results, raw PN samples (S1–S3) exhibited much higher EC_50_ values of antioxidation, suggesting that the antioxidant effect of raw PN was much weaker compared with steamed PN samples. PN steamed at 120°C showed general lower EC_50_ values than samples steamed at lower temperatures, suggesting that higher steaming temperature was related to stronger antioxidation activity of PN.

### Uncovering bioactive constituents by MLRA and PLSR

#### MLRA

The relationship between the fifteen independent variables *X*_1_, *X*_2_, *X*_3_,…, *X*_15_ (the values of normalized peak areas) and the dependent variable *Y* of each activity was established by fitting a linear equation to observed data with multiple linear regression model. The regression equations of anticoagulation and antioxidation were shown as follows:$$

(8) $$\begin{array}{l} Y_{anticoagulation}=0.1169X_{1}+0.4951X_{2}+0.4944X_{3}-0.0215X_{4}\\ \;+\;0.2931X_{5}+0.2705X_{6}+1.1867X_{7}\\ \;-\;0.03339X_{8}-0.0156X_{9}+0.2570X_{10}\\ \;+\;0.1194X_{11}-1.1664X_{12}-0.1659X_{13}\\ \;\begin{array}{c} +\;0.0703X_{14}-0.0537X_{15} \end{array} \end{array}$$

(9) $$\begin{array}{l} Y_{antioxidation}=0.2554X_{1}+1.6819X_{2}+0.3616X_{3}+0.0366X_{4}\\ \;+\;0.2698X_{5}-0.0970X_{6}+0.7760X_{7}-0.3817X_{8}\\ \;-\;0.3081X_{9}+0.6332X_{10}-0.4016X_{11}\\ \;-\;0.2679X_{12}+0.3291X_{13}-0.3322X_{14}\\ \;\begin{array}{c} -\;0.8992X_{15} \end{array} \end{array}$$

where *Y*_*anticoagulation*_ was the EC_50_ of lg [prolongation rate of *PT* (%)], *Y*_*antioxidation*_ was the EC_50_ of hydroxyl radicals scavenging activity, *X*_1_–*X*_15_ were the normalized peak areas of peaks 1–15 (Figure 1), respectively. The *F*-values for the two equations were 8.82 and 13.16, respectively. And the corresponding *P*-values were <0.01 (*R*^2^ = 0.9996), and <0.05 (*R*^2^ = 0.9999), respectively, showing that the established MLRA models were satisfied. According to the equations, EC_50_ values of a new PN sample could be obtained by inputting the corresponding peak areas to preliminarily evaluate the anticoagulant and antioxidant activities of this sample.

For the anticoagulant activity, values of Pr > |*T*| of *X*_2_, *X*_3_, *X*_5_, *X*_6_, *X*_7_, *X*_10_, and *X*_11_ were all <0.05, suggesting that constituents corresponding to peaks 2, 3, 5, 6, 7, 10, and 11 had more important role in the anticoagulation of PN. From Table 1 and Figure 1B, peaks 2, 3, 5, 6, and 7 were observed in the chromatographic fingerprints of raw PN. Among them, only peak 5 showed an increase trend along with the increase of steaming temperature and time. For steamed PN, peaks 10 and 11 were exclusively existed in the fingerprints. Thus, constituents corresponding to peaks 2, 3, 6, and 7 might play the major role in the anticoagulation of raw PN, whereas constituents corresponding to peaks 5, 10, and 11 could be the major active ones for the anticoagulation of steamed PN.

For the antioxidant activity, values of Pr > |T| of *X*_2_, *X*_3_, *X*_5_, *X*_7_, *X*_10_, and *X*_13_ were all <0.05, indicating that constituents corresponding to peaks 2, 3, 5, 7, 10, and 13 had more significant influence on the scavenging activity of hydroxyl radicals. From Table 1 and Figure 1B, peaks 2, 3, 5, and 7 were observed in the chromatographic fingerprints of raw PN. And peaks 10 and 13 are exclusively existed in the fingerprints of steamed PN. The area of peak 5 was increased along with the increase of steaming temperature and time. Therefore, constituents corresponding to peaks 2, 3, and 7 had the major role in the antioxidation of raw PN, whereas constituents corresponding to peaks 5, 10, and 13 were the major active ones for the antioxidation of steamed PN. The variations in the contents and contribution degrees of above constituents to the activities of PN may lead to the difference in the anticoagulant and antioxidant effects of raw and steamed PN samples.

#### PLSR

The PLSR models to correlate chromatographic data and the activities of PN were constructed with the 18 batches of PN samples. Since the total number of samples (18) was small and since the prediction for new samples was not our first concern, no division was made into a calibration set to build a PLSR model and a test set to validate the predictive properties. Our main concern was to focus on the indication of anticoagulant and antioxidant peaks from the modeling results. PLSR models were built from the normalized data matrix *X* containing the 18 PN fingerprints and the response matrix *Y*, i.e., either the EC_50_ of lg [prolongation rate of *PT* (%)] or the EC_50_ of hydroxyl radicals scavenging activity.

**Figure 4:**
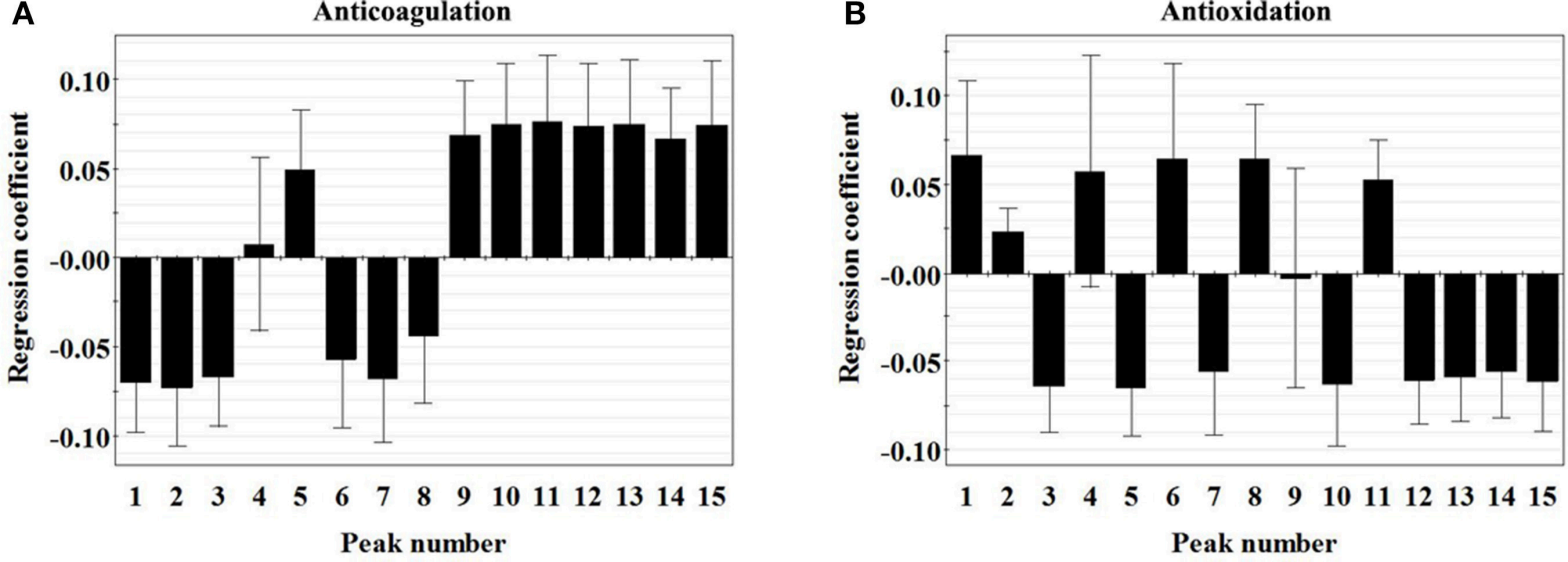
Regression coefficient plots of the anticoagulation **(A)** and antioxidation **(B)** of 15 peaks in the chromatograms of *Panax notoginseng* roots.

For the anticoagulation model, two principle components were achieved, accounting for an explained variance of 89.9% for *X* variable, 84.3% for *Y* variable, and a predictive ability (*Q*^*2*^) of 85.3% (Table S1), indicating the obtained model was excellent. As shown in the regression coefficients plot (Figure 4A), peaks 4, 5, and 9–15 were positively correlated with the EC_50_ of lg [prolongation rate of *PT* (%)], whereas peaks 1–3 and 6–8 were negatively correlated with the EC_50_ value. It should be noted that the predicted EC_50_ values could not be defined if these variables increased or decreased, because a negative coefficient did not necessarily mean that the relevant variable has the opposite effect on the anticoagulant activity. Besides, the importance of the *X*-variables for the model could be summarized by variable importance for the projection (VIP) values (usually with a threshold >1.0). Thus, constituents corresponding to peaks 1, 2, 5, 10, and 11, of which the VIP values were >1.0 (Table S2) with high absolute values of coefficients were considered to be highly related to the anticoagulant activity of PN samples.

For the antioxidant model, two principle components were achieved, accounting for an explained variance of 89.9% for *X* variable, 83.1% for *Y* variable, and a predictive ability (*Q*^2^) of 65.8% (Table S1), indicating the obtained model was excellent. As shown in the regression coefficients plot (Figure 4B), peaks 1, 2, 4, 6, 8, and 11 were positively correlated with the EC_50_ of lg [prolongation rate of *PT* (%)], whereas peaks 3, 5, 7, 9, 10, and 12–15 were negatively correlated with the EC_50_ value. Besides, the VIP value of each peak was shown in Table S2. Therefore, constituents corresponding to peaks 3, 5, 10, and 13, of which the VIP values were >1.0 with high absolute values of coefficients were considered to be highly related to the antioxidant activity of PN samples.

#### Identification of bioactive constituents corresponding to predicted peaks

**Figure 5:**
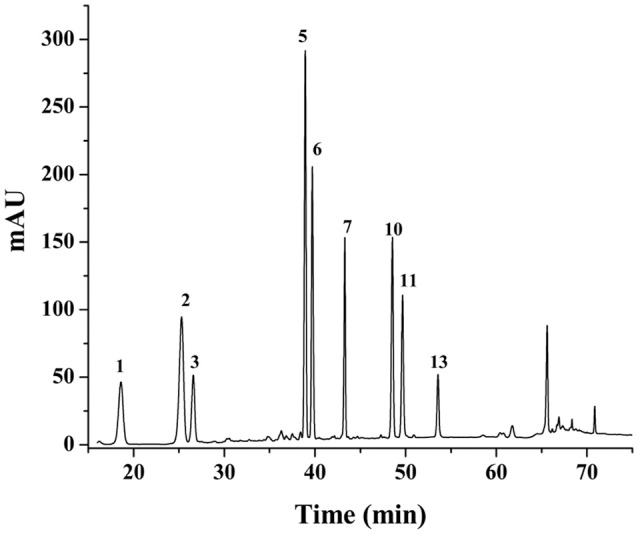
The chromatograms of the mixture of reference substances. Peak 1, 2, 3, 5, 6, 7, 10, 11, and 13 correspond to notoginsenoside R_1_, ginsenosides Rg_1_, Re, Rh_1_, Rb_1_, Rd, Rk_3_, Rh_4_, and 20(*R*)-Rg_3_, respectively.

Based on MLRA and PLSR results, constituents corresponding to peaks 1, 2, 3, 5, 6, 7, 10, and 11 were predicted to be anticoagulant ones of PN, whereas those corresponding to peaks 2, 3, 5, 7, 10, and 13 were antioxidant constituents of PN. By comparing the chromatograms of PN samples to that of the mixture of reference substances (Figure 5), peaks 1, 2, 3, 5, 6, 7, 10, 11, and 13 were identified as notoginsenoside R_1_, ginsenosides Rg_1_, Re, Rh_1_, Rb_1_, Rd, Rk_3_, Rh_4_, and 20(*R*)-Rg_3_, respectively.

From Table 1 and Figure 1B, peaks 1, 2, 3, 5, 6, and 7 were observed in the chromatographic fingerprints of raw PN. Among them, only peak 5 showed an increase trend, whereas other ones were decreased along with the increase of steaming temperature and time. And peaks 10, 11, and 13 were exclusively existed in the fingerprints of steamed PN. Thus, constituents corresponding to peaks 1, 2, 3, 6, and 7 (i.e., notoginsenoside R_1_, ginsenosides Rg_1_, Re, Rb_1_, and Rd) might play the major role in the activities of raw PN, whereas constituents corresponding to peaks 5, 10, 11, and 13 (i.e., ginsenosides Rh_1_, Rk_3_, Rh_4_, and 20(*R*)-Rg_3_) could be the major active ones of steamed PN.

**Figure 6:**
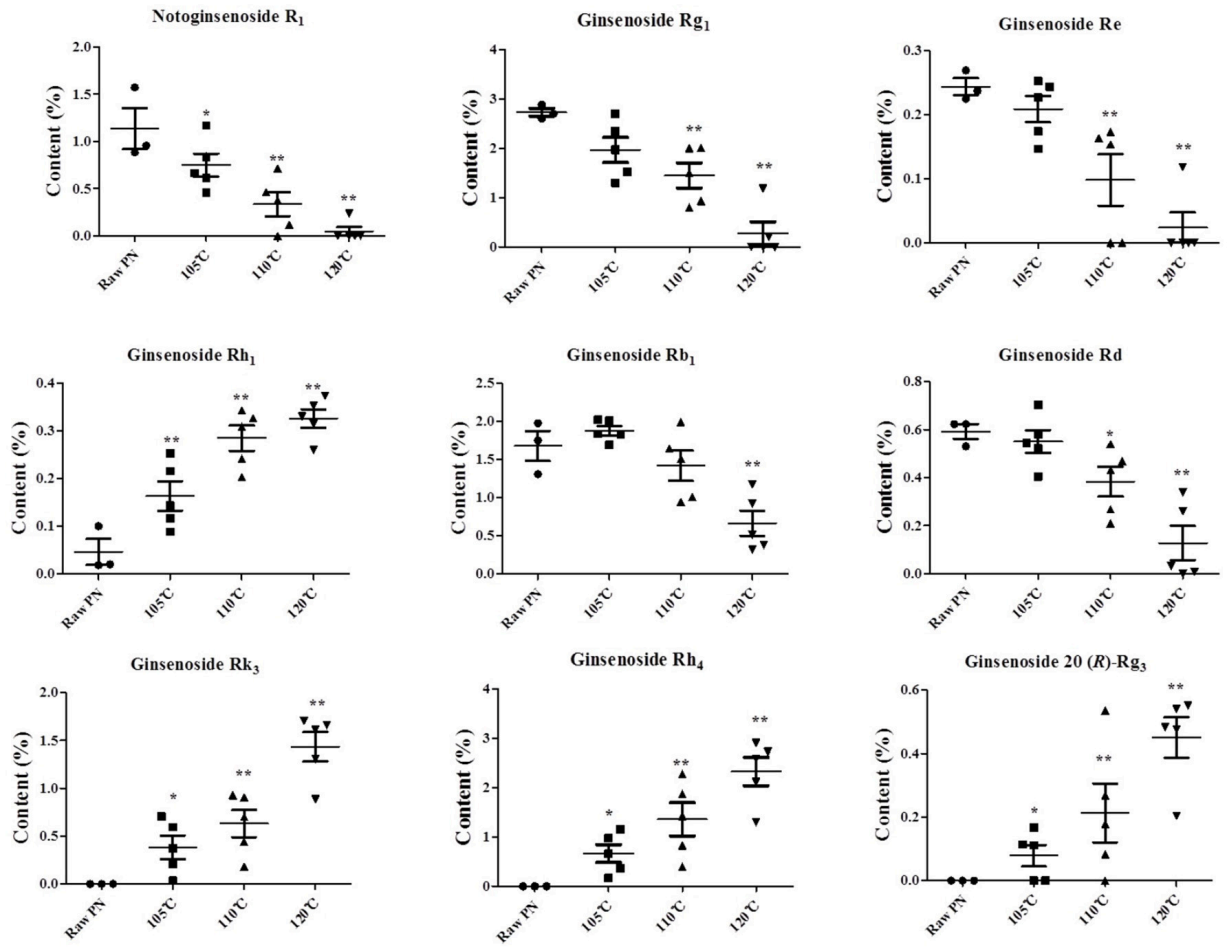
The contents of notoginsenoside R_1_, ginsenosides Rg_1_, Re, Rh_1_, Rb_1_, Rd, Rk_3_, Rh_4_, and 20 (*R*)-Rg_3_ in raw and steamed *Panax notoginseng* (PN) roots at different steaming temperatures. The points on the scatter plots represent the quantities of the nine constituents in 18 batches of PN samples. Error bars represent mean ± s.e.m. From a two-tailed unpaired Student's *t*-test, ^*^P < 0.05, ^**^P < 0.01.

Next, the contents of those constituents in raw and steamed PN were determined, as shown in Figure 6. The contents of all the constituents were significantly different between the raw PN and PN samples steamed at 120°C, and could be markers for the QC of raw and steamed PN.

### Pharmacological verification

**Figure 7:**
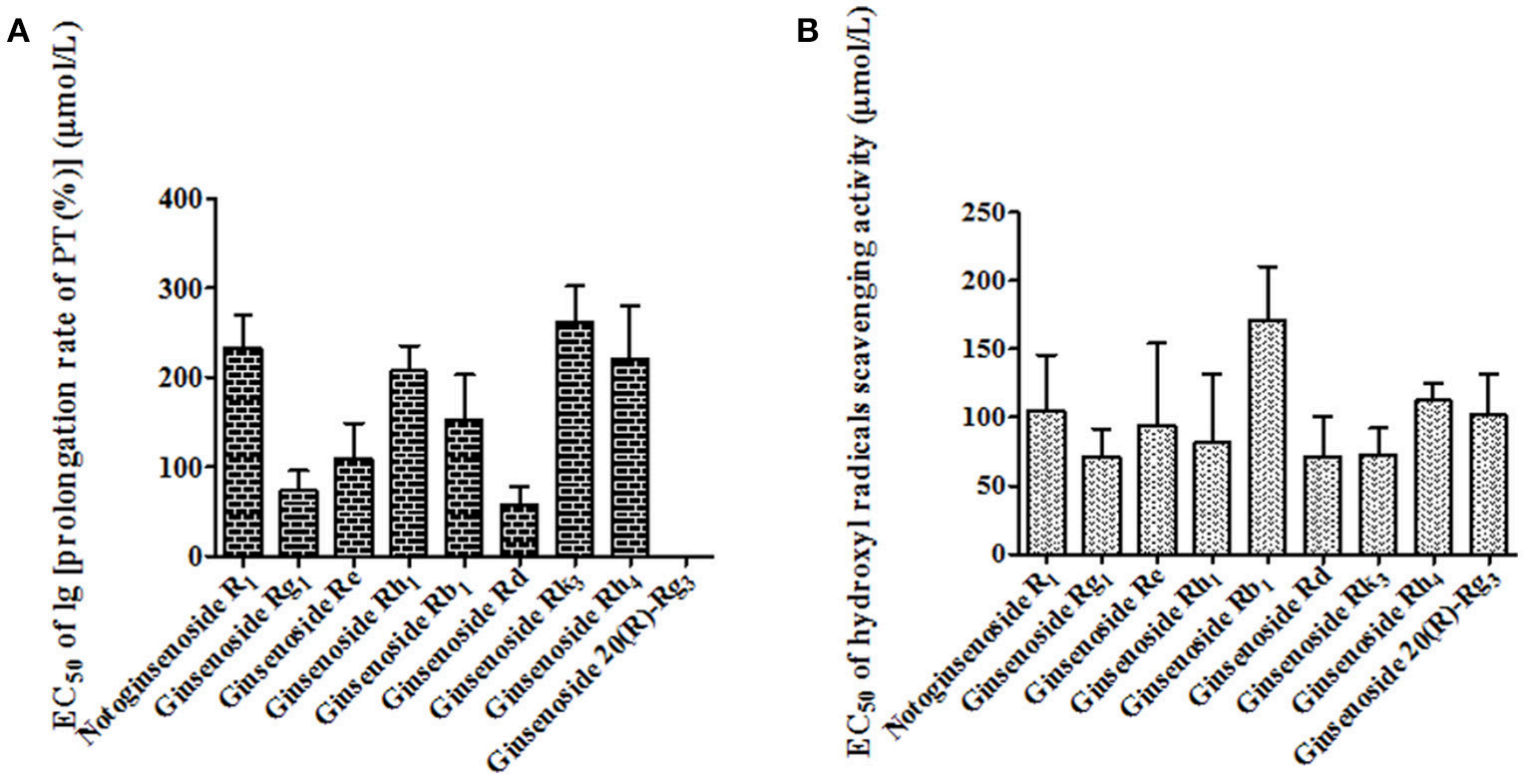
The measured EC_50_ values of anticoagulation **(A)** and antioxidation **(B)** of nine constituents in *Panax notoginseng* roots.

In order to verify the predicted active constituents and determine their contributions to each activity, the anticoagulation and antioxidation of the nine identified constituents were tested. As shown in Figure 7A, the sequence of the anticoagulant activity of the constituents was ginsenoside Rd > ginsenoside Rg_1_ > ginsenoside Re > ginsenoside Rb_1_ > ginsenoside Rh_1_ > ginsenoside Rh_4_ > notoginsenoside R_1_ > ginsenoside Rk_3_ > 20 (*R*)-Rg_3_. Meanwhile, Figure 7B showed that the antioxidant activity in descending order was ginsenoside Rg_1_ > ginsenoside Rd > ginsenoside Rk_3_ > ginsenoside Rh_1_ > ginsenoside Re > ginsenoside 20 (*R*)-Rg_3_ > notoginsenoside R_1_ > ginsenoside Rh_4_ > ginsenoside Rb_1_. According to the results, ginsenosides Rd, Rg_1_, Re, and Rb_1_, and notoginsenoside R_1_, with stronger anticoagulant activities than other constituents, and higher levels in raw PN than steamed ones, were the major active constituents for the anticoagulation of raw PN, which was consistent with the predicted result of chemometrics analyses. Among the five constituents, Rg_1_, Rd, and Re also showed certain degrees of antioxidant activity, which should be the major antioxidant constituents of raw PN. Conversely, the levels of the three constituents were decreased in PN along with the increase of steaming temperature and duration of time. And other constituents of ginsenosides Rk_3_, Rh_1_, 20 (*R*)-Rg_3_, and Rh_4_ with higher levels or exclusively existed in steamed PN should have more important role in the activities of steamed PN.

## Discussion

PN is widely used as a herbal medicine or food tonic in the global market. In order to control the quality of PN, several constituents including notoginsenoside R_1_, and some or all of ginsenosides Rg_1_, Re, Rb_1_, and Rd are determined as markers in the quality standards of different countries or regions (British Pharmacopoeia Commission, 2014; Chinese Pharmacopoeia Commission, 2015; European Pharmacopoeia 8.0; U.S.A Herbal Medicines Compendium 1.0). PN in raw and steamed forms are considered to be different in the medicinal qualities by practitioners of Oriental medicine: the raw materials eliminate and the steamed ones tonify. The so-called “eliminate” means raw PN can move stagnant blood, promote blood circulation, stopping bleeding, and resolving swelling. And the “tonify” means steamed PN can tonify the blood, enhance the immunity and anti-aging (Ge et al., 2015; Gu et al., 2015). Besides the differentiated use of raw and steamed PN by traditional medicine practitioners, the differences in the chemical composition and pharmacologic effects between raw PN and steamed PN have also been verified by modern researches (Lau et al., 2009; Wang et al., 2012). In our research, along with the duration of steaming, some peaks in the chromatograms of raw PN were decreased, whereas other ones were increased or formed. That transformation might be due to the hydrolyzation or dehydration of constituents induced by high temperature. Besides, raw PN was found to be much better in the anticoagulation than the antioxidation compared with the steamed PN, suggesting that raw PN was more suitable to treat coagulation disorders. With the increase of steaming time and temperature, the anticoagulant activity of PN weakened and the antioxidant effect strengthened, which was consistent with the traditional description of medicinal properties of raw and steamed PN. The difference in the pharmacologic effects between raw and steamed PN could be attributed to the change in the chemical composition of PN during the steaming process. However, such differences have still not been acknowledged by national statutes, in terms of the fact that QC markers for raw and steamed PN were undifferentiated at present. This may be due to that the relationships between efficacies and specific constituents of raw and steamed PN remain ambiguous.

The typical approach to select bioactive chemical markers in herbal medicines usually involves two sequential procedures: phytochemical isolation and purification to obtain pure compounds, *in vitro* and/or *in vivo* bioactivity evaluation of the individual isolates. There are some shortfalls of this reductionist methodology, such as ignorance of the possible synergism of multiple constituents, missing of the minor components, and too much time- and labor-consumption (Jiang et al., 2010). To overcome these shortcomings, the analysis of fingerprint-effect relationship has been applied to screen characteristic constituents with bioactivity related to the efficacy of a herbal medicine. Multivariate regression techniques such as PLSR, PCR, and MLRA are often used to correlate the bioactivity of a herbal medicine and its fingerprint. Regression is a generic term for all methods intended to adjust a model to the observed data, with the purpose of quantifying the relationship between two groups of variables. The adjusted model can then be used either to describe the relationship between the two variables or to predict new variables. Among these techniques, MLRA and PLSR are frequently used to specify a linear relationship between a set of dependent variables from a large set of independent variables, especially when the sample size is small relative to the dimension of these variables (Garza-Juárez et al., 2011; Wu et al., 2015). In this study, the two chemometrics modeling methods, MLRA and PLSR, were preliminarily applied to predict bioactive constituents of raw and steamed PN. Combined with the verification of pharmacologic tests, constituents responsible to different bioeffects of PN were rapidly uncovered. According to the results, nine constituents differently distributed in raw and steamed PN were predicted to be active ones related to different activities of raw and steamed PN. Notoginsenoside R_1_, ginsenosides Rg_1_, Re, Rb_1_, and Rd were predicted by MLRA and PLSR to be the major constituents related to the anticoagulation of raw PN, which was consistent with the measured EC_50_ values of anticoagulation. Among them, ginsenosides Rg_1_, Re, and Rd also showed a certain degree of antioxidation, giving evidence for determining these constituents as QC markers of raw PN. Whereas, for steamed PN samples, we found that ginsenosides Rh_1_, Rk_3_, 20 (*R*)-Rg_3_, and Rh_4_ with higher levels or exclusively existed in them could be the major constituents contributing to the activities of steamed PN. Conversely, notoginsenoside R_1_, ginsenosides Rg_1_, Re, Rb_1_, and Rd, as QC markers of raw PN, showed little or no contents in PN steamed at higher temperatures (110 and 120°C) or for a longer time, which were samples with strong antioxidation (Table 1, Figure 3). Therefore, active constituents as markers for the QC of steamed PN should be different from raw PN, because QC markers of a herbal medicine should be correlated with its safety and efficacy (Capasso et al., 2000).

Ensuring the safety and efficacy of drugs involves multiple considerations and the quality of the drug must be fundamentally guaranteed. An important part of drug QC is to ensure consistent medical and biological effects are delivered by the same drug dosage (Busse, 2000). A differentiated QC standard of steamed PN from raw PN is necessary to ensure the accuracy and safety in clinic use. Meanwhile, the anticoagulation and antioxidation effects are just parts of the major medicinal properties of PN. For better uncovering bioactive constituents of raw and steamed PN, the relationships between chemical information and other efficacies such as anti-inflammation, hemostasis, blood-tonifying, and immunoregulation, need to be further studied.

## Conclusions

In the research, there were divergences in the chemical composition and bioactivities between raw and steamed PN based on the fingerprints and pharmacologic results. Notoginsenoside R_1_, ginsenosides Rg_1_, Re, Rb_1_, and Rd with higher levels in raw PN were verified to be its active constituents, whereas ginsenosides Rh_1_, Rk_3_, 20 (*R*)-Rg_3_, and Rh_4_ with higher levels or exclusively existed in steamed PN were found to be its active constituents. Ginsenosides Rh_1_, Rk_3_, 20 (*R*)-Rg_3_, and Rh_4_ could be used in the future as new markers for the QC of steamed PN. Future research is needed to uncover bioactive constituents related to other efficacies of raw and steamed PN.

## Author contributions

YX did the writing of paper and a part of statistical analysis; LC did the anticoagulation test and pharmacological verification as well as the statistical analysis; YH did the HPLC experiments and antioxidation test; YX and XC supervised the project. All the authors read and approve the final manuscript.

### Conflict of interest statement

The authors declare that the research was conducted in the absence of any commercial or financial relationships that could be construed as a potential conflict of interest.

## Footnotes

^1^European Pharmacopoeia, 8.0, *Notoginseng root*.

^2^U.S.A Herbal Medicines Compendium 1.0, *Panax notoginseng* Root and Rhizome.
