# Supplementary material for: Uncovering Active Constituents Responsible for Different Activities of Raw and Steamed Panax notoginseng Roots
Source: Front Pharmacol. 2017 Oct 18;8:745. doi: 10.3389/fphar.2017.00745 (PMC5651511; doi:10.3389/fphar.2017.00745)
Supplement: Supplementary file 1 [file Table1.DOCX]

Supplementary Material

Uncovering active constituents responsible for different activities of raw and steamed *Panax notoginseng* roots

**Yin Xiong ^1, 2, 3^, Lijuan Chen ^1, 2, 3^, Yupiao Hu ^1,2, 3^, Xiuming Cui ^1,2, 3*^**

*** Correspondence:**

Corresponding Author: Xiuming Cui

Email: sanqi37@vip.sina.com

# Supplementary Tables

**Table S1** **Factor cumulative explained variance of PLS**

| Activities | Principal component | X variable | Cumulative X variable | Y variable | Cumulative Y variable | *Q^2^*/cum | *R^2^* |
| --- | --- | --- | --- | --- | --- | --- | --- |
| Anticoagulation | 1 | 0.693 | 0.693 | 0.791 | 0.791 | 0.763 | 0.689 |
|  | 2 | 0.206 | 0.899 | 0.052 | 0.843 | 0.853 | 0.662 |
| Antioxidation | 1 | 0.693 | 0.693 | 0.459 | 0.459 | 0.593 | 0.466 |
|  | 2 | 0.206 | 0.899 | 0.372 | 0.831 | 0.658 | 0.413 |

**Table S2 Variable importance in Projection of PLS**

| VIP | Elution sequence | | | | | | | | | | | | | | |
| --- | --- | --- | --- | --- | --- | --- | --- | --- | --- | --- | --- | --- | --- | --- | --- |
|  | 1 | 2 | 3 | 4 | 5 | 6 | 7 | 8 | 9 | 10 | 11 | 12 | 13 | 14 | 15 |
| Anticoagulation | 1.141 | 1.278 | 0.988 | 0.376 | 1.036 | 0.917 | 1.319 | 0.647 | 0.514 | 1.112 | 1.147 | 0.499 | 0.570 | 0.615 | 0.643 |
| Antioxidation | 0.407 | 0.913 | 1.130 | 0.678 | 1.142 | 0.047 | 1.051 | 0.279 | 0.468 | 1.010 | 0.956 | 0.990 | 1.065 | 0.913 | 0.981 |

# Supplementary Figures

#
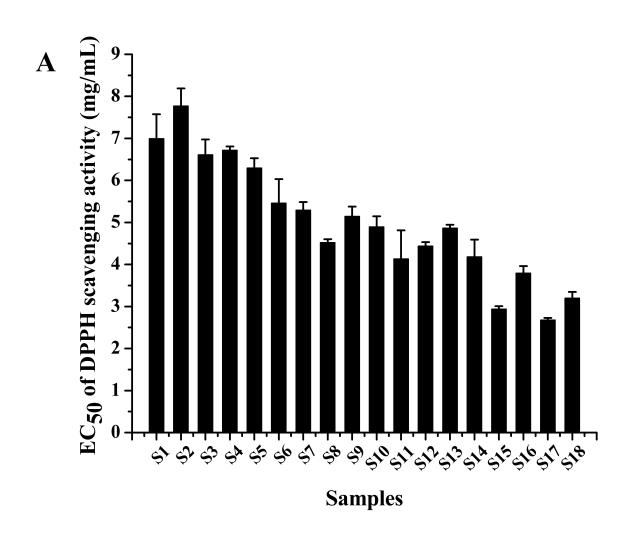

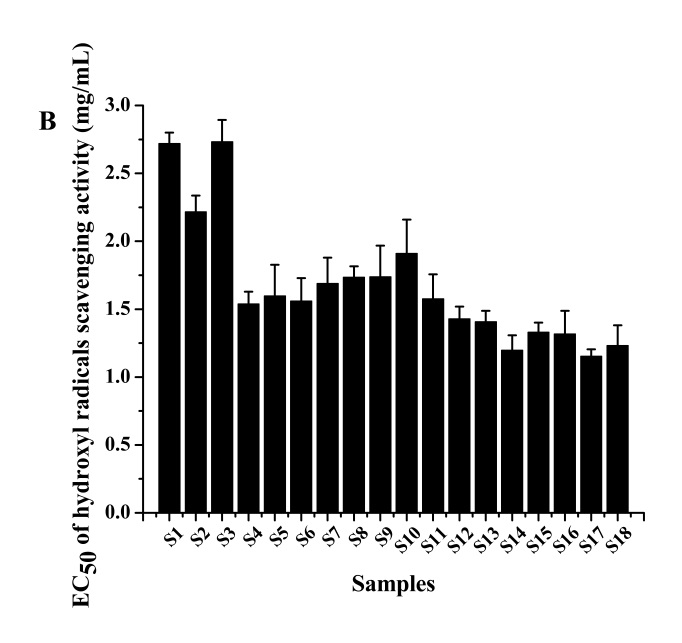


**Figure S1** DPPH (A) and hydroxyl radical (B) scavenging activities of 18 batches of *Panax notoginseng* samples.
